# Impact of Thyroid Autoimmunity on the Clinical and Biochemical Characteristics of Type 1 Diabetes Mellitus Patients

**DOI:** 10.7759/cureus.62307

**Published:** 2024-06-13

**Authors:** Ahmad Alam, Surya Kumar Singh, Ritesh Kumar, Raihan Mannan

**Affiliations:** 1 Endocrinology, Diabetes, and Metabolism, Rajiv Gandhi Centre for Diabetes and Endocrinology, Jawaharlal Nehru Medical College, Aligarh Muslim University, Aligarh, IND; 2 Endocrinology, Diabetes, and Metabolism, Institute of Medical Sciences, Banaras Hindu University, Varanasi, IND; 3 Physiology, All India Institute of Medical Sciences, Patna, IND

**Keywords:** gada, autoimmune thyroid disease, tpoab, thyroid peroxidase antibody, type 1 diabetes mellitus (t1dm)

## Abstract

Introduction: Type 1 diabetes mellitus (T1DM) is frequently associated with other autoimmune disorders that are characterized by the presence of organ-specific autoantibodies. Autoimmune thyroid disease (AIT) is the most frequent autoimmune disorder associated with T1DM. Thyroid peroxidase antibodies (TPOAb) serve as a marker for diagnosing AIT. Prior research indicates that thyroid dysfunction can negatively impact linear growth and glycemic control in subjects with T1DM. The present study was done to determine the impact of thyroid autoimmunity on the clinical and biochemical characteristics of patients with newly diagnosed T1DM.

Methods: In this single-center, hospital-based, observational cross-sectional study, we enrolled 70 patients with newly diagnosed T1DM ≤18 years of age. Type 1 diabetes mellitus was diagnosed based on the acute onset of osmotic symptoms with or without diabetic ketoacidosis (DKA), severe hyperglycemia (blood glucose >13.9 mmol/l (>250 mg/dl)), and insulin requirement from the onset of diabetes. Secondary diabetes, pancreatic diabetes (Type 3c), and maturity-onset diabetes of the young (MODY) were excluded. Participants were screened for AIT disease using TPOAb testing. Based on the presence or absence of TPOAb, the participants were categorized into two groups: Group A comprised individuals with T1DM who tested positive for TPOAb, while Group B consisted of those who tested negative for TPOAb.

Results: Out of 70 patients, 41.4% were girls and 58.6% were boys, with a mean age of 9.8±4.4 years. The prevalence of TPOAb among the cohort was 18.6%. A significant majority of patients (71.4%), presented with DKA. Group A showed significantly lower mean height standard deviation scores (SDS) compared to Group B (-0.3±0.6 vs. -0.8±0.5, p = 0.004), but no differences in weight SDS or BMI SDS. Hemoglobin A1C (HbA1c) levels, C-peptide levels, and frequency of DKA did not differ between groups. Group A had higher mean thyroid-stimulating hormone (TSH) levels (4.8±3.7 µU/ml vs. 2.6±1.5 µU/ml, p = 0.001) and a greater proportion of patients with TSH levels above the upper limit of normal compared to Group B (38.4% vs. 7.1%, p = 0.008). Additionally, Group A exhibited a higher frequency of glutamic acid decarboxylase antibody (GADA) positivity compared to Group B (46.1% vs. 17.5%, p = 0.04).

Conclusion: Patients positive for TPOAb exhibited significantly lower height SDS compared to TPOAb-negative patients. Additionally, T1DM patients with TPOAb positivity showed an increased frequency of GADA compared to those without TPOAb. However, no significant differences were found in HbA1c levels, C-peptide levels, or hematological parameters between TPOAb-positive and TPOAb-negative patients. These findings emphasize the impact of TPOAb on growth parameters in T1DM and advocate for routine screening of TPOAb in all T1DM patients, starting at the time of diabetes diagnosis.

## Introduction

Type 1 diabetes mellitus (T1DM) is frequently associated with other autoimmune disorders, such as autoimmune thyroid disease (AIT), celiac disease (CD), pernicious anemia, and Addison’s disease [1, 2]. Autoimmune thyroid disease is the most frequent autoimmune disorder associated with T1DM. Previous studies have reported that 16%-30% of T1DM subjects have AIT [3-7]. The presence of thyroid peroxidase antibodies (TPOAb) is a marker for AIT and indicates an ongoing autoimmune attack on the thyroid gland. The clinical implications of TPOAb positivity in T1DM patients are significant, as it can lead to thyroid dysfunction, which further complicates diabetes management. Thyroid dysfunction in children can have a negative impact on linear growth and glycemic control [8, 9]. While some studies suggest that subclinical hypothyroidism may adversely affect glycemic control, others have found no significant differences in hemoglobin A1C (HbA1c) levels between TPOAb-positive and TPOAb-negative patients [9, 10].

Given these potential complications, screening for TPOAb in newly diagnosed T1DM patients is recommended as part of routine clinical practice [11]. Early identification of thyroid autoimmunity can prevent complications associated with delayed diagnosis of these disorders. 

In this study, we aimed to assess the prevalence of TPOAb in newly diagnosed T1DM patients and to evaluate the impact of thyroid autoimmunity on their clinical and biochemical characteristics. By examining these associations, we hope to contribute to the existing body of knowledge and provide insights that can improve clinical practice and patient management.

## Materials and methods

Ethics approval

The study received approval from the Institutional Ethics Committee of the Institute of Medical Sciences, Banaras Hindu University, Varanasi, India (approval number: 2020/EC/2150). Informed consent was obtained from the parents of all participants, as the study enrolled patients aged 18 years or younger.

Study design

We conducted a single-center, hospital-based, observational cross-sectional study of newly diagnosed T1DM patients, three to 18 years of age attending the endocrinology clinic and emergency of the University Hospital, Institute of Medical Sciences, Banaras Hindu University, Varanasi, India, between January 2020 and March 2022. Type 1 DM was diagnosed based on the acute onset of osmotic symptoms with or without ketoacidosis, severe hyperglycemia (blood glucose >13.9 mmol/l (>250 mg/dl)), and insulin requirement from the onset of diabetes. Secondary diabetes, pancreatic diabetes (Type 3c), and maturity-onset diabetes of the young (MODY) were excluded through a comprehensive medical history and physical examination.

Participants were screened for AIT disease using TPOAb testing. Based on the presence or absence of TPOAb, the participants were categorized into two groups: Group A, individuals with T1DM who tested positive for TPOAb, and Group B, individuals with T1DM who tested negative for TPOAb.

Data collection

All patients with T1DM were assessed for age, sex, height, weight, and BMI. Weight was measured using a beam balance with minimal clothing to the nearest 0.1 kg. Height was recorded using a stadiometer to the nearest 0.1 cm, with the head positioned in the Frankfurt plane. Body mass index was calculated using the formula: weight in kilograms (kg) divided by height in meters squared (kg/m²). Age and gender-matched standard deviation scores (SDS) for height, weight, and BMI were determined using the WHO growth charts for children under five years old [12] and the Indian Academy of Pediatrics (IAP) growth charts for children aged five to 18 years [13]. Diabetic ketoacidosis (DKA) was diagnosed based on the following criteria: hyperglycemia over 200 mg/dL, venous pH less than 7.3, plasma bicarbonate (HCO_3_-) less than 15 mEq/L, and the presence of ketonuria [14].

Antibody Assays

Thyroid peroxidase antibody was measured using a chemiluminescence immunoassay (CLIA) using a Beckman Coulter UniCel DXI 800 (Beckman Coulter, Brea, CA). This assay exhibited a total imprecision of <12% at a concentration of less than 0.6 IU/ml. Thyroid peroxidase antibody values of >9 IU/ml were considered positive. The glutamic acid decarboxylase antibody (GADA) was measured by the enzyme-linked immunoassay (ELISA) method using a commercial kit (Isletest-GAD; Biomerica Inc., Newport Beach, CA). The intra-assay and inter-assay coefficients of variation (CVs) were <5.4% and <4.6%, respectively. The specificity and sensitivity of the kit were 87.1% and 85.0%, respectively. A GADA value >1.05 U/mL was considered positive. 

Other Assays

Thyroid-stimulating hormone (TSH) levels were quantified using the Access TSH (3rd IS) assay, employing CLIA methodology on the Beckman Coulter UniCel DXI 800 immunoassay platform. The assay exhibits a total imprecision of ≤10% CV at concentrations greater than 0.02 μIU/mL. The manufacturer's specified normal reference range for TSH levels is 0.4-5.3 µIU/ml. C-peptide levels were measured using the Elecsys assay, employing the electrochemiluminescence immunoassay (ECLIA) methodology on the Roche Cobas immunoassay platform (F. Hoffmann-La Roche Ltd., Basel, Switzerland). The assay's lower limit of detection for C-peptide was 0.01 ng/ml, while the limit of quantitation was 0.15 ng/ml. The HbA1c levels were measured by the high-performance liquid chromatography (HPLC) ion exchange principle. Blood glucose was measured enzymatically using the glucose oxidase-peroxidase (GOD-POD) method. The investigations were done on a fully automated Mindray SAL 6000 (Shenzhen Mindray Bio-Medical Electronics Co., Ltd., Shenzhen, China).

Statistical analysis

Quantitative variables were expressed as mean±standard deviation. They were analyzed using an independent sample t-test. Qualitative variables were expressed as percentages and analyzed using the Pearson chi-square and Fischer exact tests. All the data were analyzed using IBM SPSS Statistics for Windows, version 25.0 (IBM Corp., Armonk, NY). A p-value of 0.05 was considered significant.

## Results

In our study involving 70 newly diagnosed T1DM patients, there were 29 (41.4%) girls and 41 (58.6%) boys, with a mean age of 9.8±4.4 years. The prevalence of GADA was found to be 23%. The mean HbA1c in our study cohort was 13.4±2.1%. Notably, a significant proportion of patients (71.4%) presented with DKA at onset. The clinical and biochemical characteristics of the study cohort are summarized in Table 1. 

**Table 1:** Clinical and biochemical characteristics of the study cohort SDS: standard deviation score; BMI: body mass index; TSH: thyroid-stimulating hormone; ULN: upper limit of normal; Hb: hemoglobin; MCV: mean corpuscular volume; DKA: diabetic ketoacidosis; GADA: glutamic acid decarboxylase antibody Quantitative variables are expressed as mean±SD, and qualitative variables are expressed as numbers (%).

Characteristic	Study cohort (n=70)
Age (years)	9.8±4.4
Female	29 (41.4)
Height SDS	-0.3±0.6
Weight SDS	-1.0±0.7
BMI SDS	-1.1±0.7
TSH (µU/ml)	3.0±2.3
TSH > ULN	9 (12.9)
Hb (g/dl)	12.6±1.6
MCV (fl)	84±8.2
HbA1c (%)	13.4±2.1
C-peptide (ng/ml)	0.44±0.23
DKA at presentation	50 (71.4)
GADA positive	16 (22.9)

A total of 13 (18.6%) patients were positive for TPOAb. The clinical and biochemical characteristics of T1DM subjects with TPOAb positivity (Group A) and TPOAb negativity (Group B) are summarized in Table 2. There was no association between TPOAb and the female gender. We examined the anthropometric parameters, including height, weight, and BMI SDS. None of our patients presented with short stature; however, in Group A, the mean height SDS was significantly lower compared to Group B (-0.3±0.6 vs. -0.8±0.5, p = 0.004). There were no significant differences observed in weight SDS or BMI SDS between the two groups. The mean HbA1c levels did not significantly differ between Group A and Group B (13.4±2.1% vs. 13.3±2.3%, p = 0.48). Similarly, no significant differences were found in C-peptide levels or the frequency of DKA at presentation between the two groups. We assessed hematological parameters, including hemoglobin (Hb) levels and mean corpuscular volume (MCV). There were no significant differences in Hb levels or MCV between Group A and Group B. The mean TSH level was significantly higher in Group A compared to Group B (4.8±3.7 µU/ml vs. 2.6±1.5 µU/ml, p = 0.001). Additionally, a higher percentage of patients in Group A had TSH levels above the upper limit of normal (ULN) compared to Group B (38.4% vs. 7.1%, p = 0.008). The frequency of GADA was significantly higher in Group A as compared to Group B (46.1% vs. 17.5%; p = 0.04).

**Table 2:** Comparison of clinical and biochemical characteristics between TPOAb positive and negative groups TPOAb: thyroid peroxidase antibody; SDS: standard deviation score; BMI: body mass index; TSH: thyroid-stimulating hormone; ULN: upper limit of normal; Hb: hemoglobin; MCV: mean corpuscular volume; DKA: diabetic ketoacidosis; GADA: glutamic acid decarboxylase antibody Quantitative variables are expressed as mean ± SD, and qualitative variables are expressed as numbers (%).

Characteristic	TPOAb-postive (Group A, n=13)	TPOAb-negative (Group B, n=57)	p-value
Age (years)	10.3±3.8	9.7±4.5	0.67
Female	6 (46.2)	23 (40.4)	0.46
Height SDS	-0.8±0.5	-0.2±0.6	0.004
Weight SDS	-1.3±0.4	-0.9±0.7	0.09
BMI SDS	-1.2±0.6	-1.1±0.8	0.91
TSH (µU/ml)	4.8±3.7	2.6±1.5	0.001
TSH > ULN	5 (38.4)	4 (7.1)	0.008
Hb (g/dl)	12.1±1.6	12.7±1.5	0.19
MCV (fl)	82.1±7.4	84.4±8.3	0.36
HbA1c (%)	13.7±1.4	13.3±2.3	0.48
C-peptide (ng/ml)	0.36±0.21	0.45±0.23	0.18
DKA at presentation	8 (61.5)	42 (73.7)	0.29
GADA positive	6 (46.1)	10 (17.5)	0.04

## Discussion

Type 1 DM frequently coexists with other autoimmune disorders, often detected through serological screening due to their asymptomatic nature. Autoimmune thyroid disease is the most common autoimmune disorder linked to T1DM [1, 2]. In our study, in agreement with previous studies, we found a high prevalence of TPOAb in patients with T1DM. Upon diagnosis, 18.6% of patients with T1DM tested positive for TPOAb. The prevalence of thyroid autoantibodies in children and adolescents with T1DM has shown various results depending on the patient’s age, the duration of T1DM, the difference in cut-off points for TPOAb, and the ethnicity of the study cohort [9]. Table 3 provides a comprehensive overview of the prevalence of TPOAb positivity in patients with T1DM in India, as reported in various studies [3-7].

**Table 3:** Prevalence of TPOAb positivity in patients with type 1 diabetes in India *Expressed as median (range) TPOAb: thyroid peroxidase antibodies; NA: not available

Study	Place	Number of patients	Duration of diabetes	Prevalence (%)
Shivaprasad et al., 2017 [3]	South India	258	4.9±4.6	16.7
Dayal et al., 2015 [4]	North India	123	Recent onset	18.7
Goswami et al., 2006 [5]	North India	100	Girls: 9.0 (0.1–25)^ *^; Boys: 4.0 (0.3–22)^ *^	35
Joshi et al., 2015 [6]	West India	71	N/A	29.6
Sanyal et al., 2017 [7]	East India	50	N/A	24
Present study	North India	70	Recent onset	18.6

We compared the clinical and biochemical characteristics of patients with T1DM with positive (Group A) and negative (Group B) TPOAb. Notably, patients with TPOAb exhibited lower height SDS, suggesting potential growth impairment compared to those without autoimmunity. This aligns with the study of Reghina et al., who found a significant association between TPOAb and a lower SDS for height in their cohort of 72 children with T1DM [8]. However, there were no significant differences in age at diagnosis, female gender, weight, or BMI between groups A and B. These results align with findings from Jin et al.'s study involving 190 T1DM patients [15]. However, a significant association between TPOAb and female gender has been reported in other studies [1, 16].

In our study, we found a significant difference in the number of patients with TSH levels above the ULN between the TPOAb-positive and TPOAb-negative groups. More than one-third of patients with positive TPOAb had TSH levels exceeding the ULN. Type 1 DM patients with TPOAb had significantly higher mean TSH levels than those with negative TPOAb (4.8 µU/ml vs. 2.6 µU/ml, p = 0.001). Therefore, T1DM patients with positive TPOAb should be prospectively observed and assessed clinically and biochemically (TSH and thyroxine (T4)) to identify the progression to subclinical or overt hypothyroidism and to initiate prompt treatment with levothyroxine if necessary. Engler et al. estimated the cumulative risk for overt hypothyroidism after 10 years to be 63% in patients with increased TSH and positive thyroid antibodies, compared with only 22% in those with isolated elevations of TSH [17]. Notably, TSH elevations were also found in a group of patients without thyroid autoimmunity. This may be due to conditions other than autoimmunity, i.e., iodine deficiency resulting in thyroid dysfunction.

Our study revealed no significant difference (p = 0.48) in HbA1c levels between patients positive and negative for TPOAb. This finding is consistent with previous studies by Omar et al. and Hazzaa et al., which also reported similar results [9,18]. However, contrasting findings were noted by Reghina et al., who observed poorer glycemic control and higher HbA1c levels in TPOAb-positive patients compared to those without TPOAb [8]. Additionally, Mohn et al. found that subclinical hypothyroidism can impact glycemic control [10]. Furthermore, our study did not identify differences in C-peptide values or the frequency of patients presenting with DKA between the TPOAb-positive and TPOAb-negative groups.

We also compared the prevalence of GADA in TPOAb-positive and negative patients. The frequency of GADA was higher in patients with T1DM who had TPOAb positivity compared to negative ones (46.1% vs. 17.5%; p = 0.04). Reghina et al. and Chen et al. found a similar association between TPOAb and GADA [8,19]. These observations might be explained by the fact that GAD-65 is not only found in the brain and pancreas but also the thyroid gland [20]. Currently, the clinical utility of the GADA is to confirm the autoimmune nature of diabetes. However, the association between the GADA and TPOAb can offer new clinical applications, such as the selection of children with an increased risk for thyroid dysfunction.

Our study had certain limitations. The primary limitation was the relatively small sample size. Conducted during the COVID-19 pandemic, the study faced challenges due to lockdown restrictions, limited transport, and overburdened healthcare systems, which likely prevented some cases from reaching the hospital. Additionally, the follow-up period was short, ranging from 0 to three months after T1DM diagnosis. Ongoing follow-up of this cohort is crucial to understanding the natural history of TPOAb in T1DM patients.

## Conclusions

In conclusion, we compared the clinical and biochemical characteristics of T1DM patients who were positive for TPOAb with those who were TPOAb-negative. Patients who were TPOAb-positive had significantly lower height SDS as compared to patients who were TPOAb-negative. Also in our study, patients with T1DM and TPOAb positivity had an increased frequency of GADA as compared to T1DM subjects with TPOAb. However, there was no significant difference in HbA1c, C-peptide, or hematological parameters in patients who were positive for TPOAb as compared to those who were negative. By demonstrating the consequences on growth, these data support the recommendation for regular screening of TPOAb in all patients with T1DM, commencing from the onset of diabetes.
